# Spatiotemporal downscaling of global population and income scenarios for the United States

**DOI:** 10.1371/journal.pone.0219242

**Published:** 2019-07-24

**Authors:** David N. Wear, Jeffrey P. Prestemon

**Affiliations:** Research Project Leaders, Forestry Sciences Laboratory, USDA Forest Service, Southern Research Station, Research Triangle Park, North Carolina, United States of America; University of Vermont, UNITED STATES

## Abstract

Downscaled climate projections need to be linked to downscaled projections of population and economic growth to fully develop implications for land, natural resources, and ecosystems for future scenarios. We develop an empirical spatiotemporal approach for jointly projecting population and income at the county scale in the United States that is consistent with neoclassical economic growth theory and overlapping labor markets and that accounts for labor migration and spatial spillovers. Downscaled projections generated for the five Shared Socioeconomic Pathways used to support global scenario analysis generally show growth focused around relatively few centers especially in the southeast and western regions, with some areas in the Midwest and northeast experiencing population declines. Results are consistent with economic growth theory and with historical trends in population change and convergence of per capita personal income across US counties.

## Introduction

The size and affluence of human populations directly affect the demand for and supply of ecosystem services. Land use and natural resource projections therefore require insights into the spatial distribution of human populations and activities at scales much finer than the national level [1]. For example, population density and average personal income are effective predictors of the area of land in developed uses at the county level, and they are often statistically significant variables explaining natural resource changes [2, 3]. Official population projections for the United States [4] based on demographic methods that account for cohort-specific births, deaths, and migration are currently provided only at the national scale. Similarly, the Shared Socioeconomic Pathways (SSPs) developed as an adjunct to global climate projections [5] provide alternative population/income projections only at the continental/national scale. Analysts interested in understanding how natural resource conditions could respond to economic and population changes need fine scale projections of relevant driving predictors—that is, fine scale patterns of change based on aggregate projections. Yet methods are not well developed for downscaling national economic and demographic projections to finer spatial scales.

In climate science, several alternative methods for downscaling coarse horizontal resolution (e.g., half-degree grid scale) climate projections have been developed [6, 7]. Climate downscaling approaches include two broad methods, statistical and dynamic—the former being efficient but often bringing with it restrictive assumptions about stationarity and covariability of sets of climate variables that are simultaneously downscaled, the latter sometimes relaxing these assumptions. Dynamic methods are often considered superior because they recognize “first principles” of meteorological processes and hence would be more accurate in downscaling long-run projections. On the other hand, dynamic methods require incorporation of more relationships among variables and therefore are more computationally intensive. Downscaling approaches for economic and demographic variables would also ideally be based on first principles of the joint dynamics of economic measures of interest to achieve greater accuracy in downscaling long-run projections. The objective of this paper is to develop an empirical method for projecting county-level income and population changes that accounts for covariability of local population and income, consistent with economic growth theory, and is sufficiently parsimonious to support long-run projections for broadly defined scenarios. An expectation of the method is that it generates long-run projections of income and population variables that are consistent with historical patterns of wage-driven migration at fine spatial scales.

Our modeling is set in the context of coherent socioeconomic/climate futures and specifically addresses “downscaling” of the SSP projections of population and income for climate impact/assessment modeling. The SSP projections of population and income [8,9], when paired with atmospheric emissions described as Representative Concentration Pathways (RCPs), define an integrated assessment scenario designed to provide a consistent socioeconomic-emissions-climate future for Global Circulation Models (GCM) [10]. The coherence of the SSP-RCP combination in terms of total emissions is reconciled through Integrated Assessment Models (IAM), utilizing a set of policy assumptions [11]. We downscale population and income projections for the United States defined by the five SSPs [8,9].

Previous studies that address the downscaling of socioeconomic futures do not provide fine scale projections that account for interactions between local population and income changes. For the Special Report on Emissions Scenarios (SRES [12]), Gaffin et al. [13] downscale population density and income (GDP/land area) projections to countries and then to 1/4° grid cells using proportional change rates applied to a starting population density map. Their method ties per capita personal income to population projections, thereby embodying a restrictive assumption regarding interdependency akin to methods used in statistical downscaling of climate variables. Van Vuuren et al. [14] adopt a similar approach to population downscaling and apply exogenous convergence rules to downscale to country level GDP projections. Their approach involves within-country downscaling that assumes constant per capita personal income across gridded units. The US Environmental Protection Agency pairs a spatially explicit demographic model with a model of housing density to generate downscaled population/urbanization projections (but not economic projections), also for the fourth IPCC assessment scenarios [15]. US resource assessments [16] have rescaled and extrapolated county projections, from midrange projections produced by proprietary models of economic and demographic change [17]. McKee et al. [18] develop a spatially explicit downscaling of the US Census national projections of population alone using a demographic cohort-component method with migration factors based on Internal Revenue Service records, coupled with a growth suitability measure linked to geographic features and road infrastructure. Jones and O’Neill [19] downscale population in the United States to a gridded landscape based on a population potential model that links available land and current population with population in neighboring grid cells. Adjusting contagion parameters generates a variety of spatial patterns of change, but independent of economic projections.

These previous studies simulate change at the scale of small regional units or grid cells. Modeling focused on fine scale units such as individuals’ or households’ decisions provides an alternative approach that has proved useful for evaluating effects of policy options and other mechanisms of change [20]. Because microsimulation/agent-based approaches are built from complex specifications with many variables and require calibration constraints, those approaches can complicate long-run projections because of their high input data demands, especially when nested within broader assessment models [20, 21]. We sought a parsimonious approach consistent with historical change mechanisms and patterns of growth that relies on a small number of explanatory variables that are essential to the broad SSP scenarios. These considerations support the development of a statistical model of net change for US counties, the finest scale of reporting for important socioeconomic variables.

In this study, we posit that population and income changes are interrelated, generally consistent with observed population change and per capita income convergence across locations over time [22], and that future changes follow these population-income relationships Economic growth theory posits that factors of production, including labor and capital, driven by their owners’ desires to earn more per unit of each factor, will move from locations of relative abundance where returns (earnings) are low relative to other places, to locations of relative scarcity, where returns are higher [22]. This process tends toward equalization of relative abundance and earnings across space over time. One measurable effect of convergence is that locations which today have lower income per capita will experience faster future rates of per capita income growth compared to locations which today have higher per capita income. The presence of such economic forces, embodied in the Solow model of economic growth [22] under assumptions of free or low-cost movement of factors of production and rapid diffusion of technology, implies that fine-scale allocations of population and income across space should be jointly determined in the long run. Such jointness could mean that models of downscaled population and income that recognize their joint relationship would provide more accurate projections of population and income changes at fine spatial scales compared to methods that ignore their relationship. However, previous efforts to downscale national scale population and income to counties or grid cells in the United States have not linked population and income projections at this scale in a manner consistent with economic growth theory.

Our approach to downscaling population and income change incorporates their joint relationships to quantify the spatiotemporal process of labor migration. Assuming that the strength of net migration is governed by the level of income, we employ a statistical model that allows for joint determination of county-level population and per capita income change. Observed changes are expected to be influenced by spatial spillovers (contagion) and for a set of unmeasured county-specific factors (e.g., illiquidity of housing capital, immobility of pensioners), and these potential influences are factored into the model specification. Coefficient estimates provide a means to test for a correlation between population changes and per capita incomes that would be consistent with economic growth theory, and out-of-sample forecasts provide a test of the overall fit of the modeling system. Moreover, projections provide a means to test for consistency with observed patterns of income convergence in U.S. counties, a measure of the meta-behavior of the modeling framework. While recent work has documented rising income inequality across individuals and households in the United States [23, 24], due in part to housing supply constraints [25], convergence across regions, to varying degrees and in various forms, has held up to empirical scrutiny at multiple spatial scales: across countries [26–29], US states [30–32], and US counties [33, 34].

Our approach utilizes statistical, fixed-effects panel models of population and per capita personal income (PCPI) at the county level, with data from 1970 to 2010 on a five-year time step. To embody the joint determination of population and income, we model levels of population and PCPI as reciprocal functions of lagged population and lagged PCPI. We use a fixed effects panel model that includes both location and time fixed effects as well as spatially lagged and spatio-temporally lagged explanatory variables. Location (county) fixed effects capture time-invariant local differences in population and per capita income changes across spatial units. These fixed factors capture the county level differences in unobserved variables including demographic factors such as birth and death rates and other amenity or economic factors influencing net population changes. Spatio-temporal lags account for spatially contagious exogenous economic growth processes. Time fixed effects account for nationwide shifts in the rates of population and per capita income changes between time periods. The time fixed-effects parameters can be used to generate forecasts based on alternative assumptions regarding national rates of population and per capita income changes. When put into projection mode, the time fixed-effects component provides the mechanism to match the sum of county-level projections with nationwide population and income (projected population times projected per capita income) projections of SSPs. We define these two aggregate “growth rate” factors to determine total US level changes in population and per capita income at each time step while the estimated model is used without constraints to define the implied county level projections.

To select among alternative model specifications, we estimate several models and evaluate performance based on fit statistics applied to out-of-sample forecasts. Given that we cannot predict *a priori* the model specification that would work best in producing long-run projections of population and income at fine spatial scales, we average the projections from four specifications of the model for our downscaling. We test for consistency with economic growth theory using significance tests on model coefficients and evaluate historical and projected rates of income convergence using standard measures from economic growth theory: changes in the per capita income dispersion across counties, known as sigma convergence (decreasing relative dispersion across spatial units is consistent with convergence), and per capita income change rate versus per capita income level, known as beta convergence (an inverse relationship between growth rate and starting income level is consistent with convergence) [35]. Projections of county-level population and per capita income are provided for the five SSPs.

## Methods

### Model specification

Methods derive directly from the structure of the data: a county panel of observations measured at regular time intervals. Start with the general model:

$$Y_{it}=\delta Y_{it-1}+\beta X_{it}+\mu_{i}+\varepsilon_{it}$$ (1)

where *Y* is either population or PCPI, *i* indexes the county (*N* counties), *t* indexes the time period (*T* periods), *X* is a vector of *K* exogenous variables (including lagged values of the dependent variables), and *δ* and *β* are estimated coefficients, with separate equations for total population in the county and PCPI. As specified, errors are expected to vary with time and location, defining an error-components structure. Estimation can proceed by estimating the model conditionally on the error terms (μ and ε), defining a fixed-effects model, or can be estimated unconditionally, defining a random-effects model [36]. Furthermore, the fixed-effects model may be specified to account for differences across both location and time or location alone.

Choosing between fixed and random effects models generally depends on a Hausman test [37] to evaluate the null of equivalent random effects and fixed effects; rejection indicates the fixed-effects model is needed in order to generate statistically consistent estimates of parameters. However, the nature of the county-level data set indicates a fixed effects model *a priori*. That is, because the data set is exhaustive and not a sample (i.e., the sample is the population), the measured unit is unique and not a sample representative of a larger population [38, 39]. Although the fixed effects model accounts for constant locational or temporal effects, it does not account for spatial interactions between locations. Given the contagious nature of economic growth and the large survey units defined by counties, we anticipate that spatial dependence would arise within this data set. That is, spillover effects will likely give rise to some form of spatial dependence [36, 38] among endogenous variables, exogenous variables, or error terms of the Eq 1. Following Elhorst [38] we consider the expanded model:

$$Y_{it}=\delta Y_{it-1}+\rho WY_{it-1}+\propto_{iN}+X_{it}\beta +WX_{it}\theta +\mu_{i}+\varepsilon_{iN}+u_{t}$$ (2)

$$u_{t}=\lambda Wu_{t}+\varepsilon_{t}$$ (3)

The error components address location, time, and spatial effects structured by the spatial weights matrix **W**. We specify **W** as an *N* x *N* first order binary contiguity matrix with unit entries indicating a shared boundary between the referenced county pair and zero entries along the diagonal. The error term in Eq 2 anticipates spatial autocorrelation among error terms (Eq 3, λ≠0) but distinguishing between spatially weighted independent variables and spatial error terms is difficult [40]. Alternative estimation strategies include maximum likelihood methods and, to address the lagged endogenous variables, generalized methods of moments (GMM).

### Model estimation

#### Projections and downscaling

Forecasting requires an algorithm that applies Eq 2 to initial (lagged) conditions to project next period values, updates the temporally lagged population and income values along with associated spatial lags using the spatial weights matrix, and so on. Key elements for forecasting with the fixed effects formulation are estimates of the county-level fixed effects and application of time fixed effects terms. Fixed effects are not directly estimated from GMM estimates (which uses a differencing approach) but can be approximated as the average of residual terms (including time effects) across periods for each county. The value of a time fixed effect can be adjusted for simulations that generate US-level projections that are consistent with a desired (e.g., SSP) target level.

Downscaling the Shared Socioeconomic Pathways using our county-level model requires first selecting models among the alternatives and then developing a strategy for defining time fixed effects values for future periods that match SSP projections at the national level. As described in the Results section, model estimates indicate strong support for the specification of variables (the logarithmically transformed population equation and the untransformed PCPI equation) but less clarity regarding the specification of temporal and spatial lag structures. Because AIC statistics indicate that several alternative models are informative (have predictive power out of sample), we adopted a model averaging approach. We selected the best performing equations (based on out-of-sample goodness of fit statistics) for population and PCPI with and without spatial lags, and defined four models based on the permutations of the equations. Model averages define the projections for each scenario.

We match our projections to national SSP projections generated by the IIASA Integrated Assessment Model [41]. We directly compare rates of change for total population projections. However, comparisons of income projections are less direct. For our projections, we calculate the total US personal income by multiplying projected population by projected PCPI for each county and then sum across all counties. We compare rate of change in projected total personal income with the SSP projected rate of change for GDP—i.e., we assume that personal income remains a constant proportion of GDP, which is generally consistent with national economic projection models including the Annual Energy Outlook from Department of Energy [42]. The assumption is further supported by national income accounts, which indicate that the ratio of personal income to GDP ranged from 0.83 to 0.88 between Q2:2007 and Q2:2017, without a discernable trend in the ratio [43]. Because we do not project values for counties in Alaska or Hawaii, we match the rates of change of conterminous US projections of both population and personal income/GDP with the IIASA projections, which cover all 50 states, US territories, US Virgin Islands, and Puerto Rico.

Projections are generated on a five-year time step, consistent with estimation data, and we compare projections with SSP results by computing the residual sum of squares and mean absolute percentage error (MAPE) for five-year time steps from 2015 to 2070. We use a gridded search strategy to calibrate the models, starting with an initial naive extrapolation and then adjusting the time fixed effects parameters for the population and PCPI equations. Then, simulations are conducted for a grid of adjustment factors for both equations. From among the grid, we select models that provide the best fit for the individual SSPs based on MAPE statistics. We then repeat the grid search for each of these initial selections at each time step for progressively smaller increments in the adjustments and continue the search until the MAPE is less than 1 percent for both variables in all time periods of the projection.

#### Income convergence measures

We further examine projections for consistency with convergence growth theory. Historical and projected patterns of inter-county convergence are evaluated using trends of PCPI dispersion (sigma convergence) and the relationship between county-level economic growth and output levels (beta convergence). An approximation of β-convergence is defined by regressing the PCPI growth rate on the logarithm of base year PCPI [26]:

$$\log\left(\frac{y_{it}}{y_{it-5}}\right)=a-b\;\log\left(y_{i,t-5}\right)+\mu_{i}$$ [4

where $b=\left(1-e^{-\beta }\right)$ and β is a periodic growth rate. A positive β indicates an inverse relationship between income growth and starting PCPI levels, so that lower PCPI areas would grow at higher rates, consistent with convergence. Changes in β over time indicate acceleration (increasing β) or deceleration (decreasing β). Sigma convergence is indicated by a decline in income dispersion over time, measured as the coefficient of variation (CV) of county PCPI across counties. Spatial patterns of sigma convergence are estimated by mapping changes in the coefficient of variation for individual counties with all their neighbors (S1 and S2 Figs defined by the spatial weight index **W** and weighted by county population).

## Data

We use decadal and five-year updates to US Census estimates of total population and total real personal income in 2005 dollars from the Department of Commerce. Total personal income includes earnings plus dividends, interest income, rental income, government transfer payments less contributions to social insurance. Average personal income is estimated as total personal income divided by total population within a county.

Data were compiled for the counties of the 48 conterminous US states, with some adjustments to account for special geographic regions and changes in county names and the Federal Information Processing Standards (FIPS) codes that are commonly used by governments in data reporting for counties. Many Virginia cities exist outside of surrounding county entities, and we used the aggregation scheme of the Woods and Poole data set [17] to define aggregate county-city units in Virginia. County structure in 1970 is retained for consistency across the estimation period, by reaggregating each county formed during the estimation period with the county from which it was derived (Cibola and Valencia Counties in New Mexico, Menominee and Shawano counties in Wisconsin, Broomfield and Boulder counties in Colorado). Our dataset accounted for counties that were renamed and renumbered during the estimation period (e.g., Shannon to Oglala Lakota County, SD).

With the adjusted set of county FIPS entities, we modified a standard county-FIPS shapefile of the 48 conterminous US States to construct a spatial weights matrix (**W**) using the poly2nb and nb2listw functions in R [44]. The poly2nb function constructs the neighborhood list using one of two criteria: “Queen” for polygons with any shared boundary point and “Rook” for those with two or more shared boundary points. We compare models developed using both criteria and find a slight preference for the Queen configuration based on out-of-sample predictions. We eliminated three isolated counties (where the respective row in **W** contains only zeros) because they were physical islands and hence could not be included in the modeling. These were Dukes and Nantucket Counties in Massachusetts and San Juan County in Washington. The resulting dataset has 3073 observational units.

## Results

Econometric equations for population and per capita income indicate significant coefficients with anticipated signs and magnitudes for temporally and spatially lagged values (see SI). Estimates are consistent with posited wage-driven population migration—i.e., population growth is significantly and positively related to lagged per capita income levels—and with spatial contagion or spillover effects, as indicated by significant and positive coefficients for spatio-temporally lagged dependent variables. Alternative models were estimated using equations specified as levels or logarithms and with or without quadratic terms and then compared using various fit statistics for an out-of-sample forecast. Out-of-sample performance indicated no clear preference for a single model specification, so four informative alternatives are averaged to develop projections.

Initial model estimates addressed the potential for dynamic panel bias arising from incorporation of the lagged dependent variable (*Y*_*t*-1_) GMM estimation [45, 46]. While tests indicated no problems with residual autocorrelation, results of Sargan tests indicated universal rejection of the hypothesis of over-identifying instruments. In addition, projections based on the estimated GMM models led to explosive growth in population, which seems implausible given historical evidence. As a result, we use Maximum Likelihood estimation in lieu of GMM to estimate the panel models, accepting the potential for some bias in coefficient estimates. We also dropped the spatial autocorrelation component of the error term, accepting the loss of efficiency that may result.

Alternative models based on specifications of the error term were estimated using the PLM package in R [47]. Several alternative models are possible given the error components involved, various lag structures, and alternative specifications (i.e., logarithmically transformed versus untransformed specifications). We evaluate alternative models using coefficient estimates, fit statistics, and out-of-sample forecasts. With *T* time periods in the data set, we estimate the model for observations through period *T*-1 and then use predictions of the out-of-sample year *T* to evaluate forecast performance based on the Akaike Information Criterion (AIC), root mean square error, mean error, and mean absolute error statistics. Given our focus on projections, all variables on the right hand side of Eq 2 are expressed as temporal lags. For both equations, explanatory variables include temporally lagged (up to two lags) population and PCPI, along with spatially lagged values of the temporally lagged variables (one temporal lag only).

Eight alternative fixed effects panel models for each of the two dependent variables (population and PCPI) were estimated using maximum likelihood. These were defined as permutations of: 1) specification in levels or logarithms, 2) one or two temporal lags, and 3) with or without the spatial lag terms (S1 and S2 Tables contain coefficient estimates and evaluation statistics for all models of population and PCPI, respectively). S3 Table shows the Akaike Information Criterion (AIC), root mean squared error (RMSE) (level and percent), mean error (ME, also known as bias) (level and percent), and mean absolute error (MAE) (level and percent) for the out-of-sample (2015) evaluation.

Across the eight models for population, only two coefficients were not significant at the one percent level; both were for the lagged PCPI in the levels specification (S1 Table). Model comparisons based on out-of-sample predictions (S3 Table) indicate that a logarithmic specification for population would be preferred over a non-logarithmic model (i.e., lower RMSE, mean error (bias), and AIC values). Specification in levels yields much higher ME across these models. The inclusion of spatial lags reduces bias somewhat, but their inclusion acts to increase RMSE. For all model specifications (log/level, with and without spatial lags), the 2-lag structure outperforms a 1-lag structure.

Across the eight models for PCPI, only lagged population in one model (levels, 2-lag, spatial) and the spatially lagged PCPI in two models (level, spatial for both 1-lag and 2-lag) were insignificant (S2 Table). Model comparisons (S3 Table) indicate that the levels specifications outperform comparable models in logarithms (smaller RMSE, ME, and MAE). However, these models generate very little variation in evaluation statistics among models specified in logarithms or levels. The “levels, 1-lag, no spatial lags” model results in minimal AIC, RMSE, ME, and MAE. Among models with spatial lags, the “levels-two lag” model generates fit statistics that are the lowest among models tested.

Our ensemble of four projection models are defined by permutations of two population and two PCPI equations (see S3 Table) and projections are defined as the unweighted average of predictions from the four models. To test model predictions we conducted out-of-sample projections for the final two time steps (2010 and 2015) based on equations estimated with data through 2005. Table 1 shows mean average and absolute mean average errors of county projections for validation runs with time fixed factors held at their 2005 levels (a naïve extrapolation).The mean percent error and mean absolute percent error for 2010 are 0.3% and 3.1% respectively. As expected, these values increase to 2.3% and 5.1% respectively for 2015, but indicate an overall strong fit with little systematic bias. Spatial patterns of errors (not shown) indicate some overestimation of population in counties in Michigan and northern Ohio where economic growth was below national averages and underestimation of population in counties in Plains States that have experienced rapid economic growth due to oil exploration and development. The error patterns observed for 2015 might also reflect the somewhat temporary effects of the 2007–2009 economic recession, which hit some states and counties harder than other counties. The pattern therefore might in part constitute a shock unlikely to persist and perhaps not possible to predict at the county scale.

**Table 1:** Validation statistics. Fit statistics for out-of-sample population projections (number of people in thousands) of US Counties in 2010 and 2015. Projections generated by naïve extrapolation from equations fit with data through 2005.

Test Statistics	2010	2015
Root mean square error	11.0989	18.0460
Mean percentage error	0.2984	2.2551
Mean absolute error	2.8682	4.6745
Mean absolute percentage error	3.0975	5.1198

SSPs are organized within a two-dimensional “challenges space,” defined by challenges to mitigation and to adaptation [5], the logic being that certain economic conditions define challenges to mitigation activities and that some socioeconomic conditions provide challenges to adaptation—for example, that large populations and weak institutions limit society’s ability to adapt to climate changes and their impacts. An initial set of four SSPs describe the permutations of high and low challenges for adaptation and vulnerability, and a fifth SSP defines an intermediate case (moderate challenges to adaptation and vulnerability; [5]). Van Vuuren and Carter [48] provide a rough comparison of and crosswalk between these SSPs and SRES scenarios. Each of the five SSPs are characterized by a narrative that addresses global states of development for technology, economic growth, emissions, and institutions. Projections of key variables at global and national scales have been developed for the five core SSPs [8,9].

**Fig 1:**
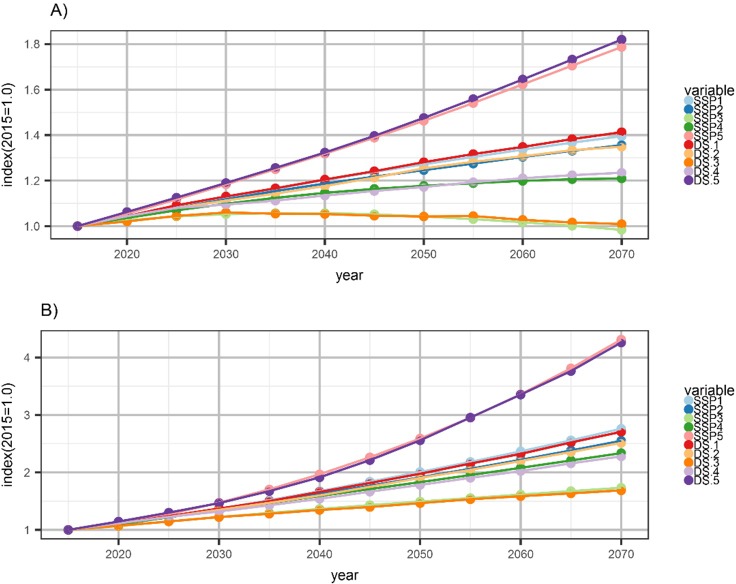
Population and GDP projections. Projections of indices of (A) US population, and (B) US Gross Domestic Product (in constant2005 dollars) projected for five Shared Socioeconomic Pathways indexed to a value of 1 in 2015. SSPx is the US projection for the referenced SSP. DS.x is the sum of the downscaled county-level projections for the referenced SSP.

SSP projections (numbered 1–5) are bracketed by SSP3, which has the lowest aggregate income (measured as Gross Domestic Product, or GDP) and population growth for the United States, and SSP5, which has the highest income and population growth for the United States. Fig 1 shows correspondence of national SSP estimates with the sum of our county projections based on the average of four projection models. Under SSP3, total US population grows slowly to a peak in 2035 and then gradually declines to 2010 population levels by 2070, while real GDP grows steadily at about 1 percent per year (from ~13 billion dollars in 2010 to ~24 billion dollars in 2070 [values in constant 2005 dollars]). Under SSP5, population expands by 86 percent (from 313 to 581 million people) between 2010 and 2070, while real GDP grows at a rate of 2.5 percent per year between 2010 and 2070, more than quadrupling over this period. SSPs 1, 2, and 4 provide intermediate projections, with population growing by between 24% and 44% (to between 390 and 451 million people) to 2070 and annual real GDP growing by between 1.4 and 1.8 percent. SSP2 provides a close match to US Census projections [4].

**Fig 2:**
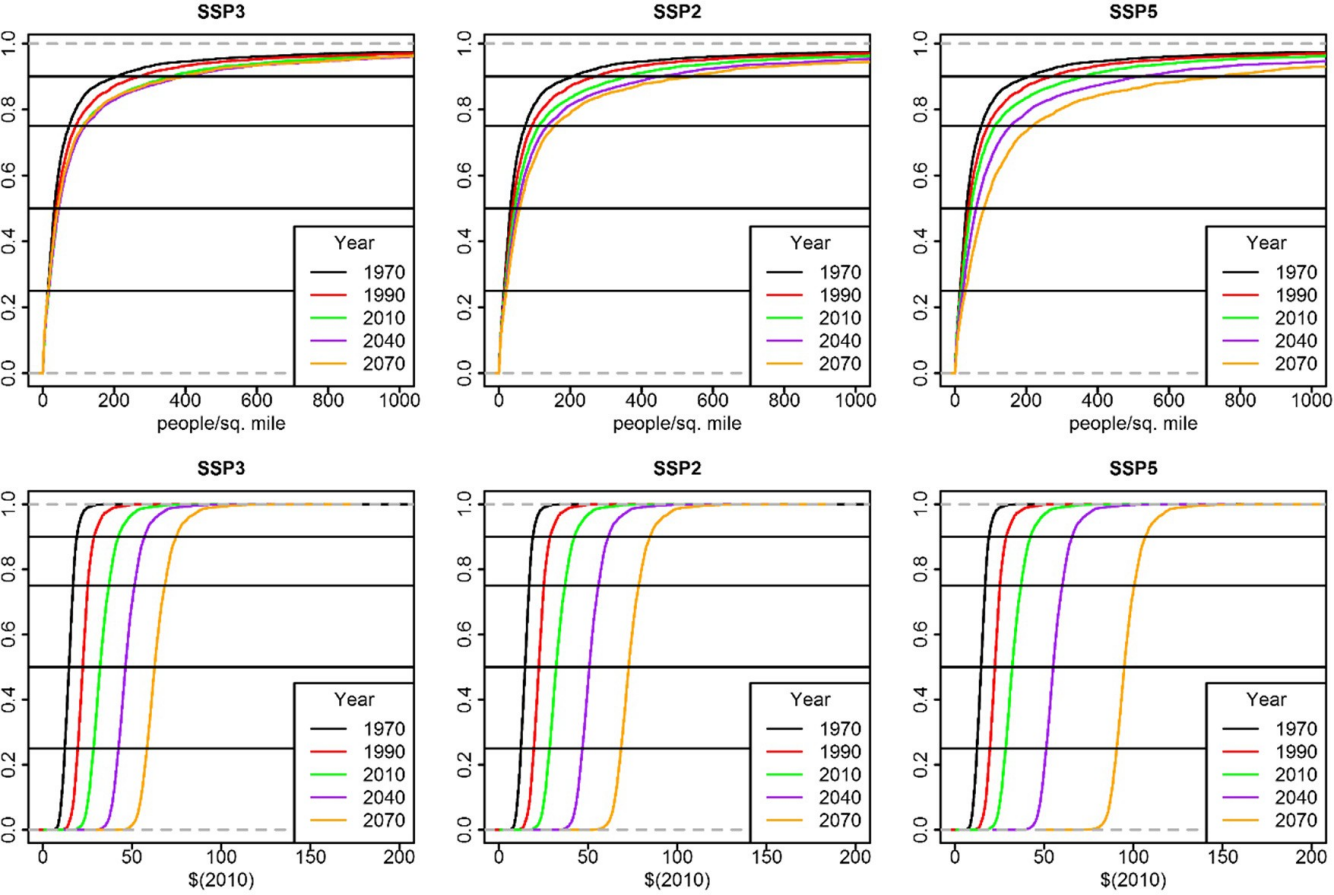
Population and per capita personal income distributions. The cumulative distribution of (A) county population density, and (B) county per capita personal income, for the United States for historical (1970, 1990, 2010) and projected (2040, 2070) years for three Shared Socioeconomic Pathways SSP3 (low growth), SSP2 (moderate growth), and SSP5 (high growth).

Projected rates of population growth are consistently higher for the most populous counties. For all SSPs, the cumulative distributions of county populations for the historical period and the projection period show outward shifts in the distribution of population, with little change in population density for counties in the lower quartile and strongest expansion in population density in the upper quartile (Fig 2A). Between 1970 and 2010, the population density for the county at the 90^th^ percentile shifted from about 200 to about 380 people per square mile (ppsm). For SSP3 (lowest population growth), the county at the 90^th^ percentile shows negligible changes to 2070. For SSP2 (moderate population growth), the county at the 90^th^ percentile density in 2070 is about 575 ppsm. For SSP5 (highest population growth), the county at the 90^th^ percentile density in 2070 is at 750 ppsm. A discernable shift in the density of the 50^th^ percentile county is indicated only for SSP5—i.e., counties below median population levels in 2010 are generally not projected to gain population for all scenarios except SSP5.

**Fig 3:**
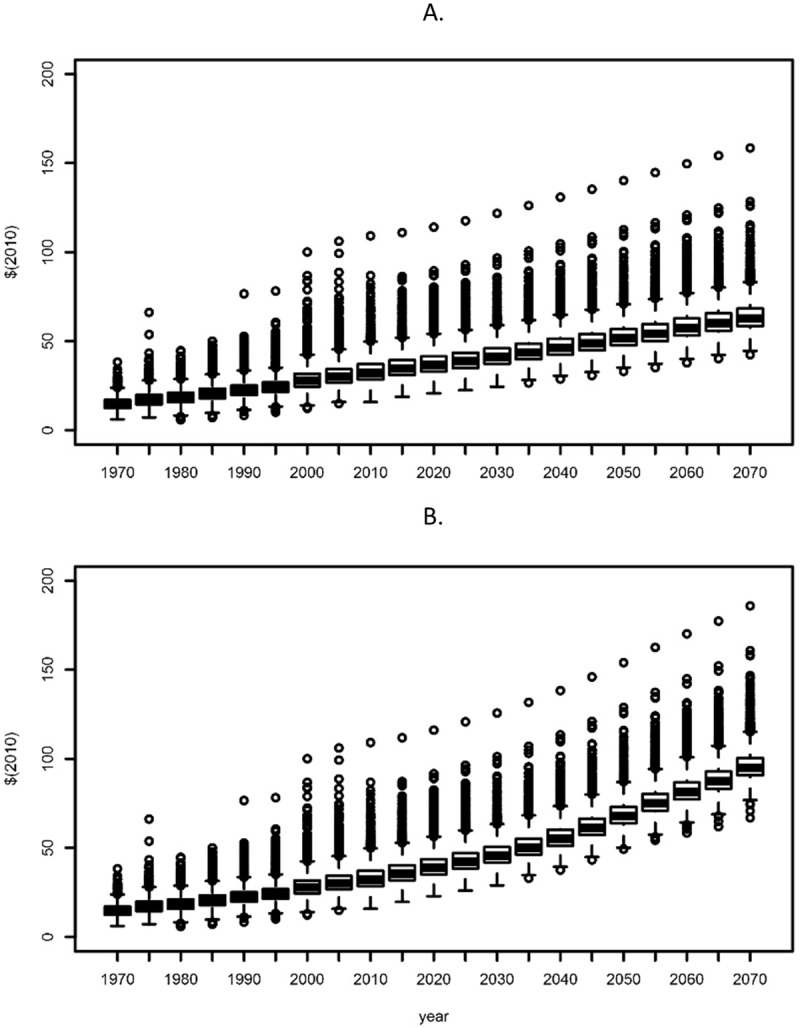
Per capita personal income by county. Boxplots of county per capita personal income for historical (1970–2010) and projected (2015–2070) periods for the SSP3 (lowest growth) and SSP5 (highest growth) scenarios. Center line represents the median income, the box represents the interquartile range (25th to 75th percentile), and the whiskers are 1.5 times the interquartile range. Individual circles indicate outliers.

The PCPI cumulative distributions shift outward in a fashion that is qualitatively distinct from the population variable. The distributions shift outward uniformly across all percentiles (Fig 2B) and are scaled by the overall growth rate of the scenario. Boxplots of historical and projected PCPI at the county level for SSP3 and SSP5 (Fig 3) show a regular increase in the median PCPI of counties, with a relatively constant interquartile range, indicating a decline in the range in percentage terms. The pattern of outliers indicates a relatively constant number of high PCPI counties (i.e., with values greater than 1.5 times the interquartile range).

**Fig 4:**
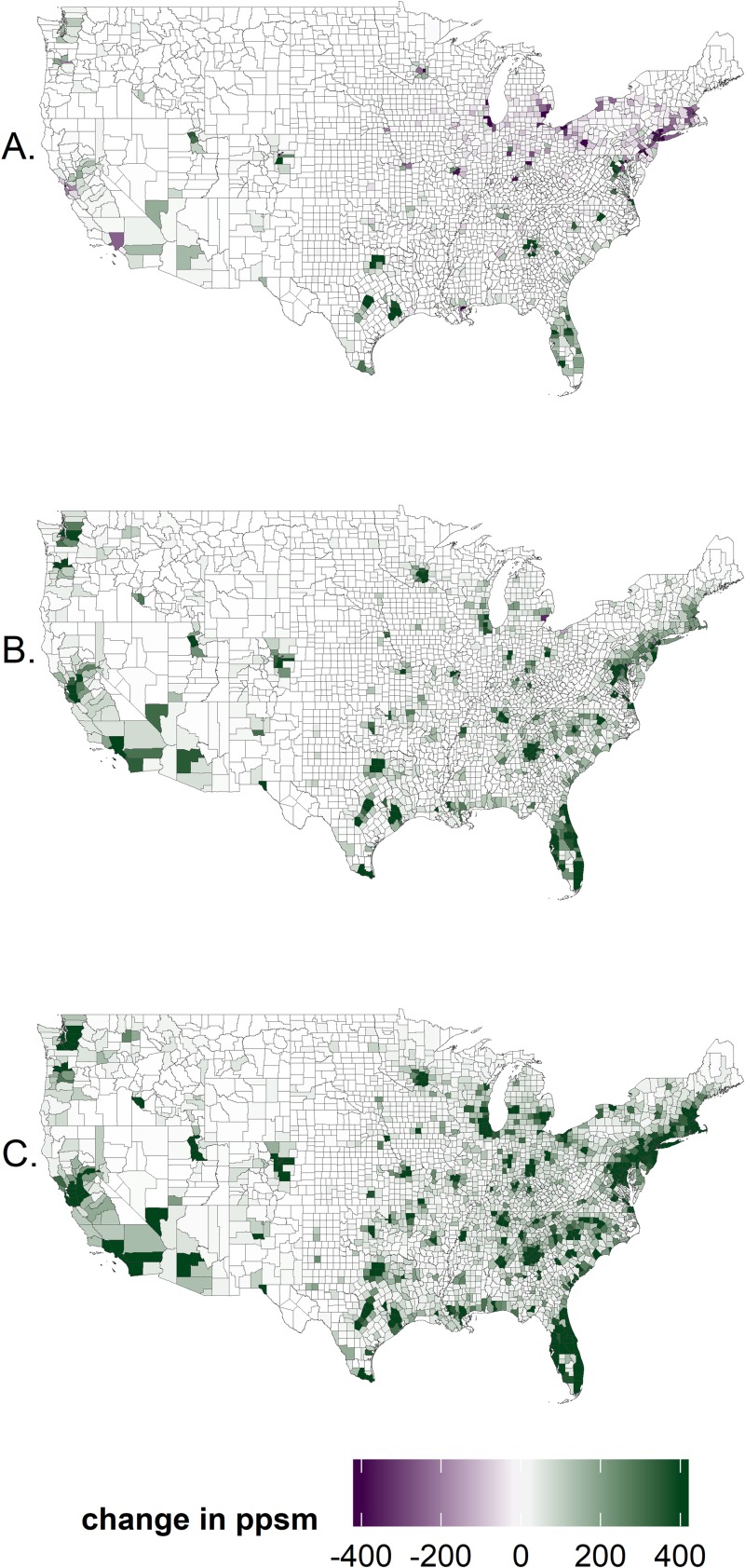
Population projections. Projected change in population density (people per square mile, ppsm) between 2010 and 2070 for three Shared Socioeconomic Pathways: (A) SSP3 (low growth), (B) SSP2 (moderate growth), and (C) SSP5 (high growth).

Maps of population change (Fig 4) show the influence of spatial factors and differences in growth across scenarios. For SSP3, populations shift spatially, in spite of little overall growth and consistent with income convergence: populations decline in rural counties and across the industrial north and Midwest but grow strongly in a few metro counties, especially in the South and West (counties containing Raleigh, Charlotte, Atlanta, Tampa, Orlando, Dallas, Houston, Austin, Denver, Salt Lake, and Seattle). For moderate growth scenarios (SSP2 in Fig 4), growth expands around these metro counties but also extends to counties of northeastern coastal areas, Midwestern cities (e.g., Chicago and Detroit), and other western cities (San Francisco, Los Angeles, and Portland). For SSP5, growth expands outward from the same growth centers found for SSP2, and no counties experience sizable population losses. Across all scenarios, the most rural areas (especially in the Great Plains, Midwest, and South) do not experience substantial growth in population. Among broad regions, the southeastern United States has the highest population growth rate for all SSPs, with only slightly lower rates for the western United States. For SSP3, the population growth rate for the remainder of the United States is negative (-13%), for the other SSPs it is 45–68% lower than the southeastern and western regions. Under SSP5 populations are projected to roughly double in the southeastern and western United States (+103% and +95% respectively) and increase by 56% in the remainder of the states.

**Fig 5:**
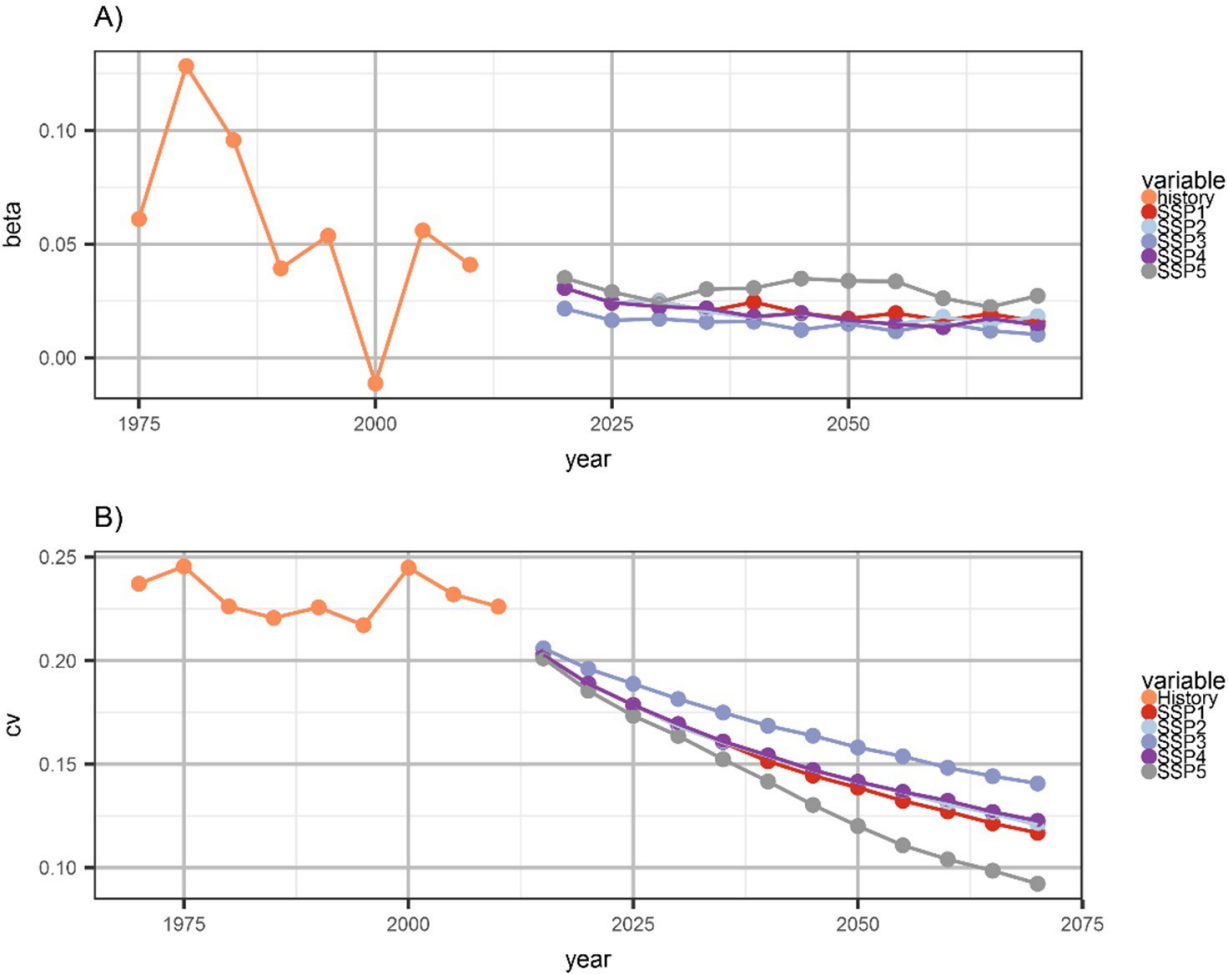
Convergence measures. (A) Beta measures of convergence, defined as the rate of personal income convergence derived from a regression of income growth rate on the logarithm of income in the base year (five year time step), and (B) sigma measures of convergence, measured as the coefficient of variation of per capita personal income across counties, for the historical period (1975–2010) and SSP projections (2015–2070).

Historical and projected patterns of inter-county income convergence are evaluated using trends of PCPI dispersion (sigma convergence) and the relationship between the county-level PCPI growth rate and PCPI levels (beta convergence; see Methods). Beta values for projections generally follow historical trends, with an ongoing but decelerating convergence indicated by a positive β that decreases over time. Historical values of β trended downward over the estimation period (Fig 5A), from an average of 0.094 between 1970 and 1985 to an average of 0.029 between 1995 and 2010 (β is negative for only one period, 1995–2000). Values of β defined by the projections are everywhere positive (p = 0.01) and range between 0.010 and 0.035, with highest values for SSP5 (the high growth scenario) and lowest values for SSP3 (the low growth scenario), implying that the rate of convergence is positively related to the economic growth rate. Projections for all SSPs indicate continued β-convergence in county PCPIs through 2070.

Trends in the coefficient of variation (CV) for projected county PCPI are consistent with the most recent (2000–2010) sigma convergence, indicated by shrinking PCPI dispersion across counties. Historical CV estimates indicate a repositioning of CV between 1995 and 2000, which aligns with the negative β value for that period (Fig 5A). Values trended downward between 1975 and 1995, rose between 1995 and 2000, and then declined again between 2000 and 2010 (Fig 5B). Projections show a continuous downward trend in CV from 2015–2070 (Fig 5B) for all SSPs, with the strongest rate of decline for SSP5 (high economic and population growth scenario) and the lowest rates for SSP3 (low economic and population growth scenario). That is, the CVs for projections of all SSPs are consistent with sigma convergence through 2070. We further note that the differences in rates of sigma convergence at the county scale in the conterminous United States mirror similar ordering (SSP3 the slowest, SSP5 the fastest) of sigma convergence implied by the SSP projections of income across countries.

Changes in the income CV for individual counties with only their neighbors (defined by a spatial weight index **W** and weighted by county population; see Methods) provide an additional spatial assessment of sigma convergence. The distribution of neighborhood CV values (see SI) indicates a shift toward spatial convergence (reduced CV’s) of PCPIs between 1970 and 1990 and a slight decrease in convergence between 1990 and 2010. Projections indicate a steady increase in spatial convergence through 2070, with spatial convergence rates directly related to overall economic growth rates (SSP3 the lowest; SSP5 the highest). Maps of CVs (see S2 Fig for SSP2) graphically indicate widespread diminution of variation between 2010 and 2040, with some notable exceptions: counties along the eastern front of the Rocky Mountains from Wyoming to New Mexico, some counties in the northern Great Plains, and counties at the periphery of high-PCPI counties along the northeastern seaboard.

## Discussion

We develop a parsimonious model of county-level population and PCPI change based on lagged values of these variables and spatial neighborhood variables using fixed effects panel models. Model average projections indicate good performance in out-of-sample forecasts. Multi-decade projections define county level change in these variables, scaled to national projections of population and total income (GDP) from the Shared Socioeconomic Pathways by adjusting the time fixed effects parameters. Adjustment of the fixed effects parameters allows us to downscale national projections of population and income in a way that extends the observed patterns of change, including spatial contagion, experienced over the past 40 years. Parsimony is a strength for projections allowing for projections with few parameters relative to detailed mechanistic demographic models where future birth, death, and migration rates would need to also be modeled. Still, the assumptions of the model would become less certain as projection length increases.

The projections to 2070 preserve the structure of adjustments reflected in the historical data (1970–2010). Historical changes are likely limited by the costly movement of cohorts of the population (based on age, income, education attributes that are not among the explanatory variables), which our statistical models quantify with a partial adjustment process and county-level fixed factors. The gradual changes captured by our model are consistent with the finding that interrelated housing market and transportation costs would impede mobility below costless migration patterns and allow local unemployment to persist [49]. As with any empirical model, we anticipate that forecast variance increases over the length of the projections—i.e., that variables embedded in the county level fixed factors whose effects are parameterized using historical data might eventually evolve to different levels.

Historical patterns of population growth indicate increasingly concentrated populations over time in the United States. Likewise, our projections indicate an ongoing process of interregional population movement, with a continued shift in the share of the nation’s population away from the Northeast/Midwest and toward the South and West. A comparison of the lowest population growth scenario (SSP3) with the highest population growth scenario (SSP5) demonstrates how strong income growth drives population expansion from existing metropolitan cores into but not much beyond the closest surrounding rural counties. For all scenarios, a large share of the current rural United States experiences either a stable or a shrinking population.

Overall, projections are consistent with recent historical (and slowing) PCPI convergence across US counties for both beta- and sigma-convergence measures. Observed and modeled growth is consistent with neoclassical growth theory, where low (high) income counties are expected to have higher (lower) growth rates. In our models, this is driven largely by the population variable, consistent with labor migration away from low income and toward high income counties, as owners of labor and capital seek higher earnings. Results indicate a direct correspondence between economic growth rates and the rate of convergence (beta measure). Addressing both income and population in the projection framework captures first principles of economics and allows for an accounting for economic growth dynamics across space. Significant spatial lag terms in the models conform to the notion of overlapping labor markets and contagious population (labor) growth that is consistent with relocation and/or expanded commuting regions [50].

The utility of our modeling approach would derive from providing an integrated set of income and population projections and a qualitatively distinct set of population projections that account for income driven labor migration. As an *ex post* test of our approach we compared the model-ensemble population projections with projections developed using a gravity-based/urbanization potential approach [51] and downscaled to a 1-km raster [52]. After summarizing data to the county level, we applied the same validation tests (Table 1) against observed data for 2010 and 2015 for these projections (see S1 Appendix). For both time steps and all test statistics, the gravity-based model generated higher errors than the model-ensemble suggesting that incorporation of income-driven migration provided improved explanatory power. Mapping error terms at the county level for the two models shows that the gravity-based model generally underestimated populations in southern and western urban centers and overestimated population in rural areas and northern urban centers, consistent with dominant patterns of population migration in the United States. Comparison of projections to the year 2070 shows similar differences in pattern (S1 Appendix).

All projection models have limitations defined by their formulations. The model is derived from a simple concept, labor-driven migration, resulting in gradual reductions in per capita income variation across space, augmented by agglomeration dynamics. Notably, this model does not account for the influence of climate change projections on potential long run change in population or economic growth. While historical climate influences are embedded in the model and might be evident in near term projections, longer term movements in populations in the United States may be structurally influenced by climate change—e.g., by sea level rise in coastal counties of the East and temperature increases in the Southwest, where our model projects that much population growth would occur. Likewise, people also migrate to seek desired amenities and higher education opportunities. A general limitation of the model is the extrapolation of fixed effects. The spatial fixed effects and spatiotemporal parameters embodied in the models capture omitted historical factors including demographic and various non-income contributions to well-being, but in projection mode the models cannot predict changes in the unmeasured variables, for example the emergence of new places with desirable amenity and education features. While a more complex formulation is possible, each additional explanatory variable must be projected within a scenario. Parsimony is a virtue for projection models, and our approach provides a transparent and replicable starting point for conducting local effects analysis of global change scenarios and for evaluating alternative projection approaches.

## Supporting information

S1 Appendix Comparing projections. (DOCX)

S1 Table Coefficient estimates for eight specifications of the population equation defined by the permutations of: Specified in levels or natural logs, one or two temporal lags, and inclusion or not of spatially lagged terms. “POP” is the population variable, “PCPI” is the per capita personal income variable.
(DOCX)

S2 Table Coefficient estimates for eight specifications of the per capita personal income equation defined by the permutations of: Specified in levels or natural logs, one or two temporal lags, and inclusion or not of spatially lagged terms. “POP” is the population variable, “PCPI” is the per capita personal income variable.
(DOCX)

S3 Table Estimates of out of sample model performance for the eight formulations of population and per capita personal income equations using seven evaluation statistics: Akaike Information Criterion (AIC), root mean square error (RMSE), mean error, and mean absolute error statistics. The latter three are presented in levels and percentage terms.
(DOCX)

S1 Fig The cumulative distribution of the coefficient of variation for counties of coterminous 48 US for historical (1970, 1990, 2010) and projected (2040, 2070) periods for three Shared Socioeconomic Pathways. (TIF)

S2 Fig Maps of the neighborhood coefficient of variation (cv) for counties for 2010 and the projected proportional change in cv between 2010 and 2040 for SSP2. (TIF)

S3 Fig Map of prediction errors (proportion) for population projections from the NASA Socioeconomic Data and Applications Center (SEDAC) for the year 2015 (aggregated to county level). (TIF)

S4 Fig Map of prediction errors (proportion) for population projections from the estimated model ensemble for the year 2015 (county level). (TIF)

S5 Fig Map of differences in population projections from the NASA Socioeconomic Data and Applications Center (SEDAC) and the estimated model ensemble for the year 2070 (people per square mile). (TIF)

S1 Dataset Income and population data used to estimate downscaling models. (XLSX)
