# Supplementary material for: Spatiotemporal downscaling of global population and income scenarios for the United States
Source: PLoS One. 2019 Jul 24;14(7):e0219242. doi: 10.1371/journal.pone.0219242 (PMC6655633; doi:10.1371/journal.pone.0219242)
Supplement: S1 Appendix — (DOCX) [file pone.0219242.s001.docx]

# Appendix S1: Comparing Projections

The overall utility of our modeling approach would derive from providing an integrated set of income and population projections and a qualitatively distinct set of population projections that account for income-driven labor migration. As an *ex post* test of our approach we compared our population projections with SSP projections developed using a gravity-based/urbanization potential approach that does not account for interactions with income (1), downscaled to a 1-km raster (2). These downscaled projections are part of the NASA Socioeconomic Data and Applications Center (SEDAC, 3) and were accessed at: <https://sedac.ciesin.columbia.edu/data/set/popdynamics-pop-projection-ssp-downscaled-1km-2010-2100>.

After summarizing 1-km data to the county level, we applied validation tests for population estimates against observed data for 2010 and 2015 for these projections (2015 estimates were derived by averaging 2010 and 2020 estimates). For both time steps and all test statistics, the gravity-based model generated higher errors than the estimated model ensemble used here, suggesting that incorporation of income-driven migration provided improved explanatory power. Mapping error terms at the county level (Fig. S3 and Fig. S4) for the two models shows that the gravity-based model generally underestimated populations in southern and western urban centers and overestimated population in rural areas and northern urban centers, consistent with dominant patterns of population migration in the United States.

We also compared the spatially downscaled projections for SSP1 at the year 2070 (Fig. S5) and find qualitatively distinct projections. As shown in Fig. S5, when compared with the ensemble model, the SEDAC model generated much lower projections of population in the western and southern regions and higher projections of population in the northeastern region.

Table 1. Validation test statistics for population predictions from the estimated model ensemble and for downscaled SSP1 projection data from the NASA Socioeconomic Data and Applications Center (SEDAC). SEDAC estimates for 2015 are estimated as the average of 2010 and 2020 projections.

| Test Statistics | Model [2010] | SEDAC [2010] | Model [2015] | SEDAC [2015] |
| --- | --- | --- | --- | --- |
| Root mean square error | 11.0989 | 33.9136 | 18.0460 | 44.6252 |
| Mean percentage error | 0.2984 | 6.5107 | 2.2551 | 8.2257 |
| Mean absolute error | 2.8682 | 9.0267 | 4.6745 | 11.7507 |
| Mean absolute percentage error | 3.0975 | 12.1715 | 5.1198 | 14.5482 |

# References

1. Jones B, O’Neill, BC. Spatially explicit global population scenarios consistent with the Shared Socioeconomic Pathways. Environ Res Lett. 2016;11(8), p.084003.
2. Gao J. Downscaling Global Spatial Population Projections from 1/8-degree to 1-km Grid Cells. NCAR Technical Note NCAR/TN-537+STR, 2017; doi:10.5065/D60Z721H.
3. Center for International Earth Science Information Network - CIESIN - Columbia University, United Nations Food and Agriculture Programme - FAO, and Centro Internacional de Agricultura Tropical - CIAT. 2005. *Gridded Population of the World, Version 3 (GPWv3): Population Count Grid*. Palisades, NY: NASA Socioeconomic Data and Applications Center (SEDAC). <http://dx.doi.org/10.7927/H4639MPP>. Accessed 3 June 2019.
