# Supplementary material for: Spatiotemporal downscaling of global population and income scenarios for the United States
Source: PLoS One. 2019 Jul 24;14(7):e0219242. doi: 10.1371/journal.pone.0219242 (PMC6655633; doi:10.1371/journal.pone.0219242)
Supplement: S1 Table — “POP” is the population variable, “PCPI” is the per capita personal income variable. (DOCX) [file pone.0219242.s002.docx]

Table S1. Coefficient estimates for eight specifications of the population equation defined by the permutations of: specified in levels or logs, one or two temporal lags, and inclusion or not of spatially lagged terms. “POP” is the population variable, “PCPI” is the per capita personal income variable.
