# Supplementary material for: Spatiotemporal downscaling of global population and income scenarios for the United States
Source: PLoS One. 2019 Jul 24;14(7):e0219242. doi: 10.1371/journal.pone.0219242 (PMC6655633; doi:10.1371/journal.pone.0219242)
Supplement: S3 Table — The latter three are presented in levels and percentage terms. (DOCX) [file pone.0219242.s004.docx]

Table S3. Estimates of out of sample model performance for the eight formulations of population and per capita personal income equations using seven evaluation statistics: Akaike Information Criterion (AIC), root mean square error (RMSE), mean error, and mean absolute error statistics. The latter three are presented in levels and percentage terms. Selection of equations for inclusion in the projection ensemble are indicated.

| Model Specification | | | | | | | | | |
| --- | --- | --- | --- | --- | --- | --- | --- | --- | --- |
| Specification | levels | levels | logs | logs |  | levels | levels | logs | logs |
| Number of lags | 1 | 2 | 1 | 2 |  | 1 | 2 | 1 | 2 |
| Spatial lags? | yes | yes | yes | yes |  | no | no | no | no |
|  |  |  |  |  |  |  |  |  |  |
| Population equation | | | | | | | | | |
| AIC | 11,702.91 | 10,715.14 | 11,591.92 | 10,199.28 |  | 11,478.61 | 10,437.18 | 12,652.12 | 11,374.58 |
| RMSE | 6.7050 | 5.7058 | 6.5850 | 5.2464 |  | 6.4689 | 5.4570 | 7.8299 | 6.3562 |
| % RMSE | 6.4546 | 5.4927 | 6.3391 | 5.0505 |  | 6.2273 | 5.2533 | 7.5375 | 6.1188 |
| Mean error | -1.7724 | -1.4388 | -0.3139 | -0.3244 |  | -1.0553 | -0.1182 | 0.0498 | 0.0470 |
| mean % error | -18.8757 | -20.7666 | -1.6508 | -1.5206 |  | -11.3857 | -8.1462 | -1.2657 | -1.0861 |
| mean abs error | 3.2447 | 2.8404 | 2.1420 | 1.7857 |  | 2.6728 | 1.8701 | 2.2544 | 1.8417 |
| mean abs % error | 19.3474 | 21.0980 | 2.7216 | 2.4584 |  | 14.4028 | 10.0245 | 2.5226 | 2.2212 |
| Ensemble model? |  |  |  | yes |  |  |  |  | yes |
| Per capital personal income equation | | | | | | | | | |
|  | | |  |  |  |  |  |  |  |
| AIC | 10,232.99 | 10,184.9 | 11,102.13 | 11,215.16 |  | 8,768.495 | 10,173.37 | 11,145.95 | 11,244.34 |
| RMSE | 5.2787 | 5.2342 | 6.0806 | 6.1894 |  | 4.1622 | 5.2278 | 6.1281 | 6.2229 |
| % RMSE | 5.0816 | 5.0387 | 5.8535 | 5.9583 |  | 4.0068 | 5.0326 | 5.8993 | 5.9905 |
| Mean error | 3.1898 | 2.9318 | 3.9426 | 4.0578 |  | 2.2639 | 2.9053 | 3.9823 | 4.0716 |
| mean % error | 7.2455 | 6.4826 | 9.3921 | 9.7063 |  | 5.3590 | 6.4074 | 9.4903 | 9.7297 |
| mean abs error | 3.3595 | 3.2026 | 4.0760 | 4.1812 |  | 2.5479 | 3.1874 | 4.1164 | 4.2001 |
| mean abs % error | 7.8448 | 7.4301 | 9.8325 | 10.1026 |  | 6.1505 | 7.3930 | 9.9286 | 10.1373 |
| Ensemble model? |  | yes |  |  |  | yes |  |  |  |
