# Supplementary figures and images for: Spatiotemporal downscaling of global population and income scenarios for the United States
Source: PLoS One. 2019 Jul 24;14(7):e0219242. doi: 10.1371/journal.pone.0219242 (PMC6655633; doi:10.1371/journal.pone.0219242)

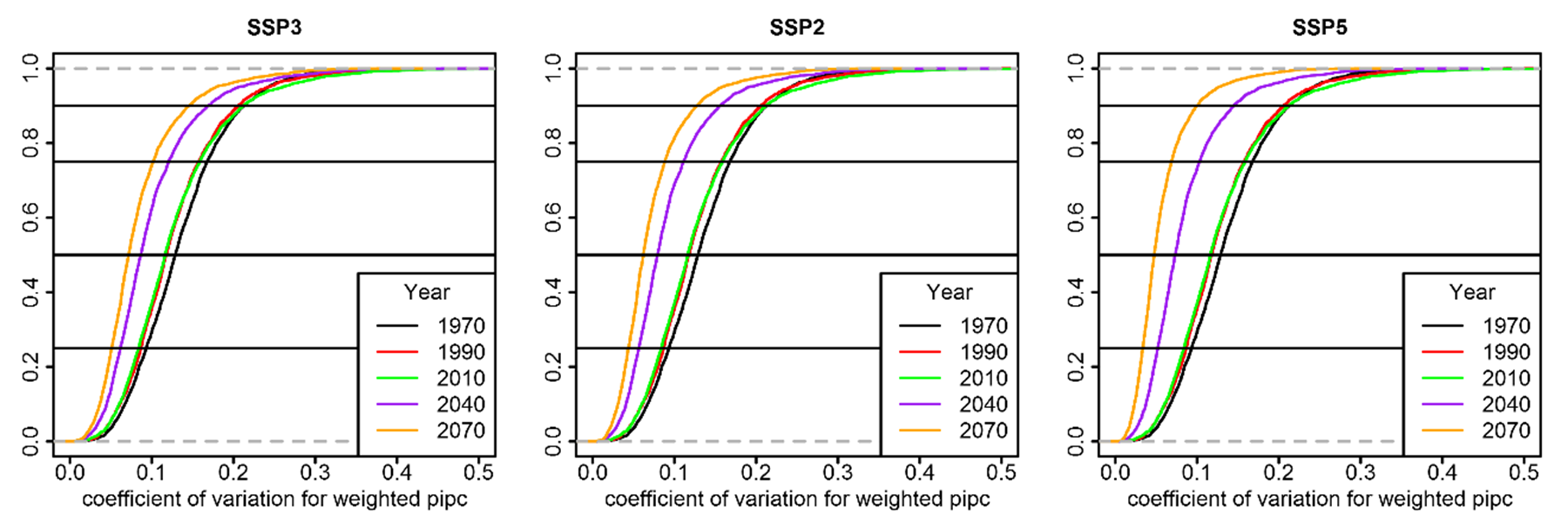

Supplement: S1 Fig — (TIF) [file pone.0219242.s005.tif]

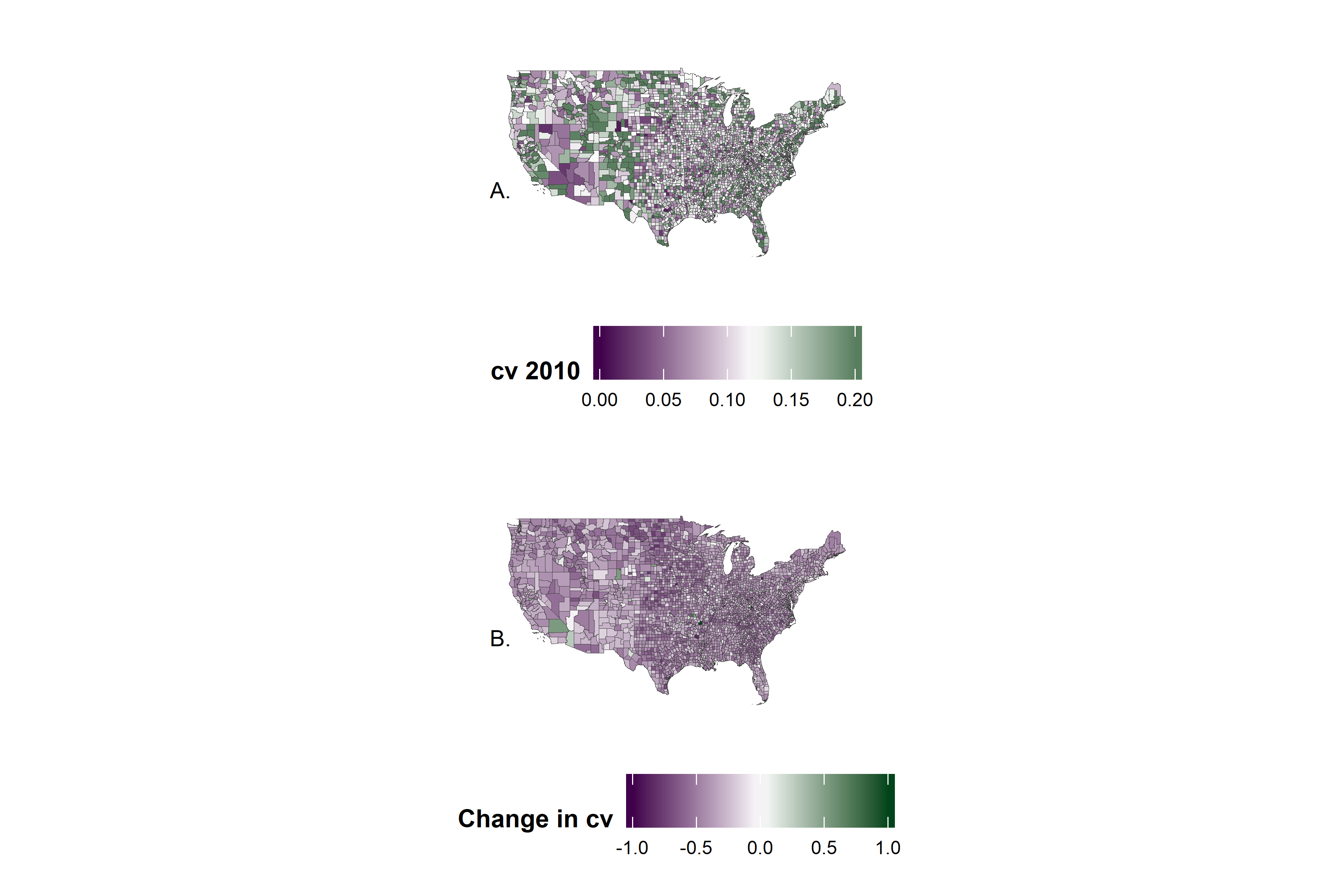

Supplement: S2 Fig — (TIF) [file pone.0219242.s006.tif]

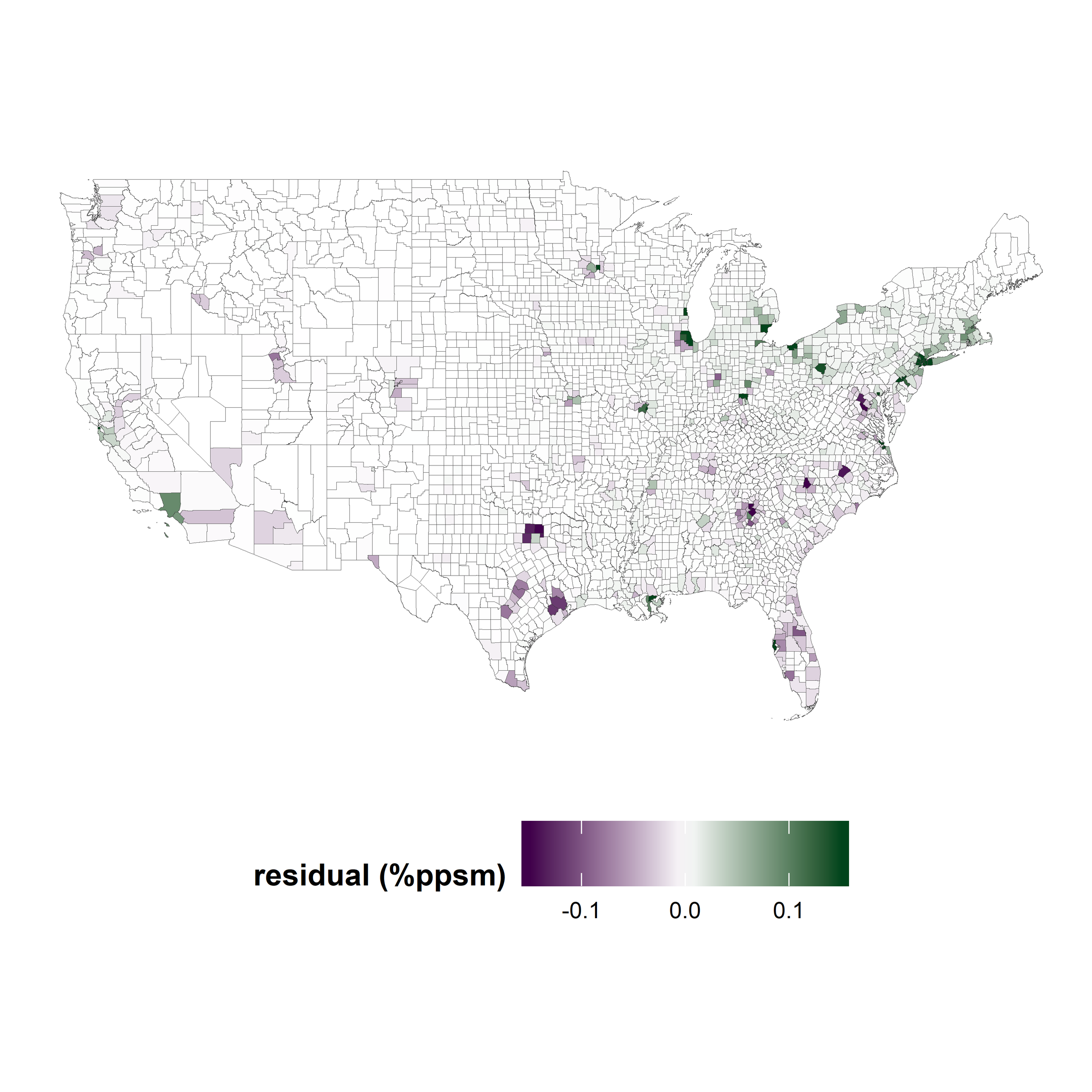

Supplement: S3 Fig — (TIF) [file pone.0219242.s007.tif]

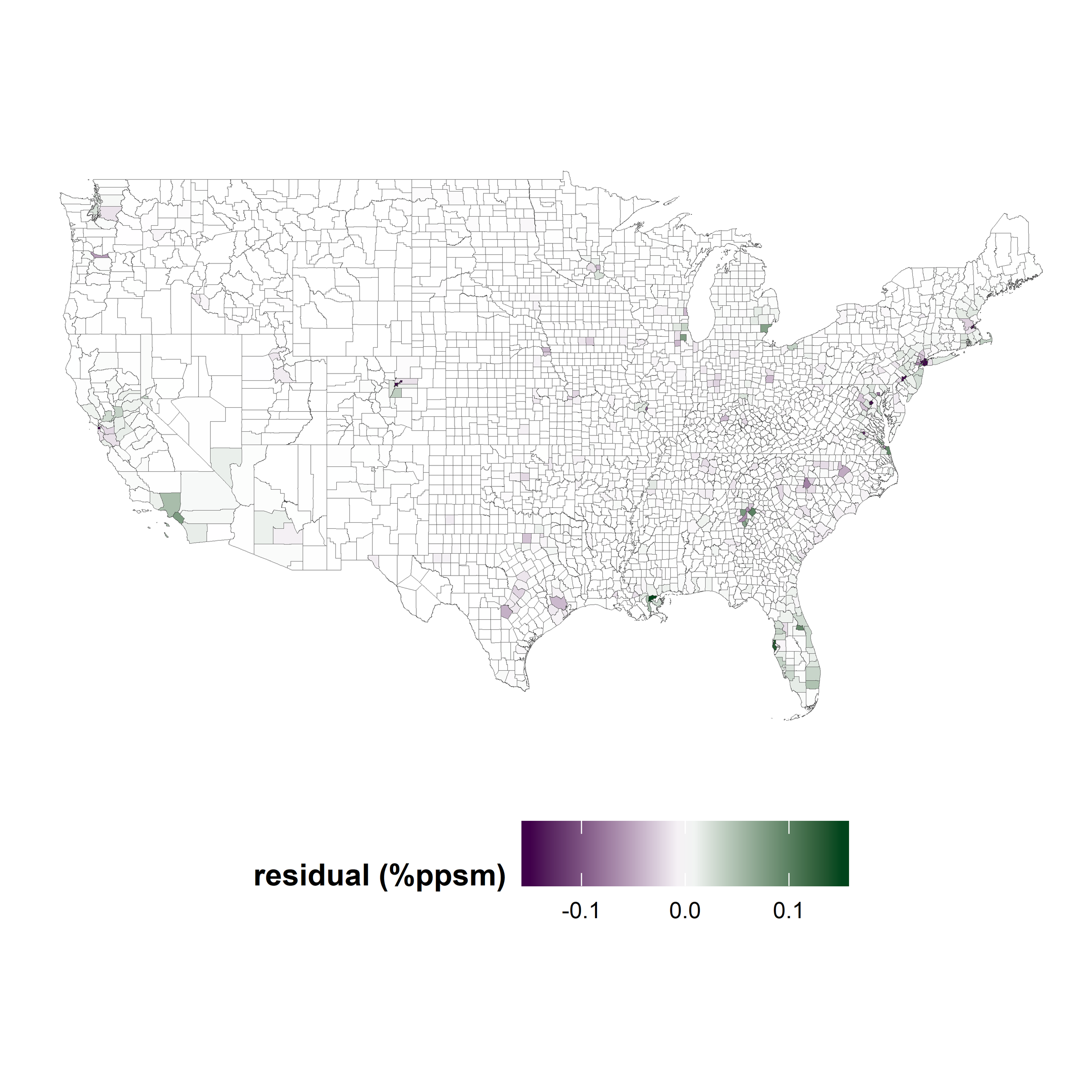

Supplement: S4 Fig — (TIF) [file pone.0219242.s008.tif]

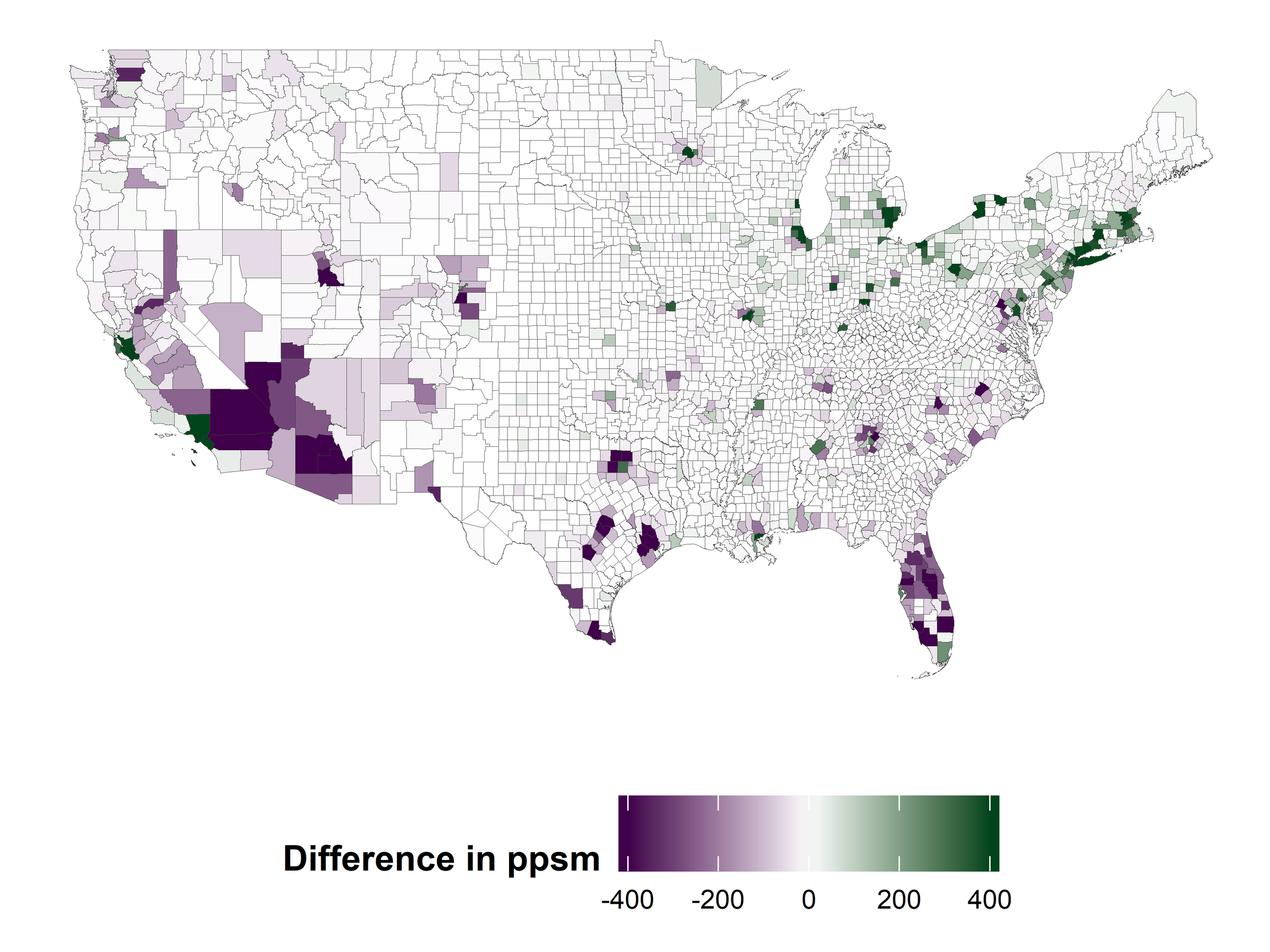

Supplement: S5 Fig — (TIF) [file pone.0219242.s009.tif]
